# Prevalence and risk factors of Occult Hepatitis C infections in blood donors from Mexico City

**DOI:** 10.1371/journal.pone.0205659

**Published:** 2018-10-19

**Authors:** María de la Luz Martínez-Rodríguez, Luis A. Uribe-Noguez, Carla I. Arroyo-Anduiza, José Antonio Mata-Marin, Gamaliel Benitez-Arvizu, María L. Portillo-López, Alicia Ocaña-Mondragón

**Affiliations:** 1 Unidad de Investigación Médica en Inmunología e Infectología, Centro Médico Nacional “La Raza”, Delegación 2 Noreste del D.F., Instituto Mexicano del Seguro Social, Mexico City, Mexico; 2 Banco Central de Sangre, Centro Médico Nacional “La Raza”, Delegación 2 Noreste del D.F., Instituto Mexicano del Seguro Social, Mexico City, Mexico; 3 Hospital de Infectología, Centro Médico Nacional “La Raza”, Delegación 2 Noreste del D.F., Instituto Mexicano del Seguro Social. Mexico City, Mexico; 4 Banco Central de Sangre, Centro Médico Nacional “Siglo XXI”, Delegación Sur, Instituto Mexicano del Seguro Social, Mexico City, Mexico; Centers for Disease Control and Prevention, UNITED STATES

## Abstract

**Background:**

The circulatory system is the main mechanism for transmission of the Hepatitis C Virus (HCV). A new class of HCV infections, Occult HCV infection (OCI), is defined as the presence of HCV-RNA in hepatocytes with the absence of HCV in the serum/plasma utilizing current laboratory assays. Different groups have reported the prevalence of OCI; however, its associated risk factors have not been established. In Mexico, there are no reports about OCI, so the objective of our study was to determine the prevalence of OCI in total blood donors in Mexico City, as well as its associated risk factors.

**Methods:**

Blood donors that were considered eligible for donation, according to NOM 253-SSA1-2012, were randomly selected. Demographic data was collected from 1,037 donors. Plasma and peripheral blood mononuclear cells were assessed for HCV-RNA. The presence of HCV-RNA was determined by nested PCR for the 5'-UTR region. Logistic regression was used to calculate the odds ratio (OR) and 95% confidence interval (95%CI) to determine the level of association.

**Results:**

The prevalence of OCI was 3.4% among blood donors. Homosexual relationships (OR = 5.52, 95%CI: 1.53–19.92, p<0.05) and acupuncture (OR = 3.56, 95%CI: 1.41–8.98, p<0.05) were significantly associated with OCI.

**Conclusion:**

There is a significant presence of OCI in the blood donor population in Mexico City. The main risk factors for OCI transmission are homosexual relationships and acupuncture. This study supports the increased use of sensitive and specific screening tests for blood bank testing.

## Introduction

Chronic Hepatitis C Virus infection (CHC) is a world health problem, affecting more than 71 million people and causing about 500,000 deaths each year. It is estimated that, in 2015, there were 1.75 million new Hepatitis C Virus (HCV) cases [1,2]. In Mexico, the prevalence of HCV infected persons is estimated to be 1.4% of the total population. Interestingly, blood transfusions are one of the most frequent risk factor for developing HCV infections [3,4].

HCV is a positive, single-stranded RNA, enveloped virus that belongs to the family *Flaviviridae*, genus *hepacivirus* [5]. The diagnosis of HCV-infections in blood banks and reference laboratories is performed by detecting antibodies against the virus and/or the presence of viral RNA in the serum or plasma [6,7]. However, this does not include examining alternative cells where the presence of HCV has been reported, such as the peripheral blood mononuclear cells (PBMCs) [8].

In 2004, Castillo and colleagues reported for the first time, in patients with CHC of unknown origin, Occult HCV infections (OCI) [9]. OCI is defined as the presence of HCV-RNA in hepatocytes or PBMCs with undetectable plasma or serum levels of anti-HCV or HCV-RNA serum utilizing current laboratory assays, regardless of hepatic transaminase elevation. Recently, the capacity of viral replication in PBMCs has been reported in OCI. This is possibly due to the presence of negative, single-strand HCV-RNA, which could favor HCV replication and potentially infectious OCI patients [8]. The prevalence of OCI has been previously reported in high-risk groups to contract HCV. For example, patients with cryptogenic liver disease, the prevalence was reported to be 74.2% [10]. In hemodialysis patients, the prevalence ranged between 3.0 to 15.1% [11]. In patients without apparent liver disease, the prevalence was reported to be 3.3% [12].

Contact with contaminated blood and other body fluids have a very important role in the transmission and propagation of HCV infections and several risk factors have been established: blood transfusions, tattoos, use of non-sterile surgical material, intravenous drug use, and acupuncture. In contrast, the associated risk factors for OCI are not entirely clear. It has been reported that being male, having a history of surgeries and dental procedures, and an age over 30 years old are predictive factors of OCI [13,14]. Only three reports, from China, Egypt, and Spain, are available that focus on the prevalence of OCI among blood donors [15–17]. In Mexico, the prevalence of OCI is unknown. Therefore, the objective of this study was to determine the prevalence of OCI as well as establish the associated risk factors for OCI in blood donors from Mexico City.

## Methods

### Study population

During the period from November 2015 to July 2016, donors were asked to participate in this multicenter, descriptive study. Blood donors came from the Banco Central de Sangre del Centro Médico Nacional "La Raza" and Banco Central de Sangre del Centro Médico Nacional "Siglo XXI", both hospitals are members of Instituto Mexicano del Seguro Social (IMSS). All participants provided written informed consent to participate in the study and responded to a questionnaire, which was specially designed to collect demographic data and to identify risk factors for OCI transmission. The study protocol conforms to the ethical guidelines of the 1975 Declaration of Helsinki as reflected in a priori approval by the IMSS Research Committee (registration number: R-2014-3502-132). We followed the guideline according to “Strengthening the Reporting of Observational studies in Epidemiology” (STROBE) criteria. The data is provided at https://figshare.com/account/home#/data (doi:org/10.6084/m9.figshare.6359318.v1).

### PBMC isolation

In four tubes with EDTA anticoagulant (BD Vacutainer K2E, Becton, Dickinson and Company, New Jersey, USA), 16-mL of blood was obtained from the collection bag of the Teruflex blood bag system (Terumo BCT, Tokyo, Japan). PBMCs were collected according to Instituto de Diagnóstico y Referencia Epidemiológicos (INDRE) [18]. The plasma fraction was isolated by centrifugation at 3,000 rpm. The PBMCs were obtained by performing three washes with 0.5 mM magnesium chloride and three washes with phosphate buffered saline pH 7.4 (PBS, Catalog number: 70011079, Gibco, Thermo-Fisher Scientific, Massachusetts, USA). The pellet was resuspended in Trizol (Invitrogen Life Technologies, Burlingron, Canada) and stored at -80 °C until analyzed. All blood components obtained from the donors were stored in the blood bank until the results of the study were obtained. Anti-HCV or HCV-RNA positive products were discarded according to Mexican regulations: NOM-087-SEMARNAT-SSA1-2002 [19].

### RNA extraction and RT-PCR

Isolation of viral RNA from plasma was performed using the QIAamp Viral RNA Mini kit (catalog number: 27106, QIAGEN, Hilden, Germany) per the manufacture’s recommendations. Isolation of viral RNA from PBMCs in Trizol was performed with the RNAeasy kit (Catalog number: 74106, QIAGEN) per the manufacture’s recommendations. As a control, PBMCs and plasma from confirmed HCV-positive and HCV-negative patients were included. RNA was recovered in 50 μL of RNase free water and quantified with the Nanodrop 2000 equipment (Thermo-Fisher Scientific, Massachusetts, USA). All samples were adjusted to a concentration of 20 ng/μL. The cDNA was obtained by RT-PCR using the AMV Reverse Transcriptase kit and random hexamer primers (Catalog number: M9004, Promega, Wisconsin, USA) following the manufacturer’s instructions.

### Nested PCR

The 5'-UTR region of HCV was amplified by nested PCR using the MyTaq DNA Polymerase kit (catalog number: BIO-21107, Bioline, London, UK). The KY78 and KY80 primers were used for the first amplification and the FIP and RIP primers for the second amplification, as described by Choo et al. [20] and certified by Holland et al. [21]. PCR conditions were as follows: initial denaturation at 95 °C for 1 min, followed by 28 cycles of 95 °C for 15 s, 55 °C for 20 s, and 72 °C for 15 s, and a final step of 72 °C for 10 min. For the second PCR, the program was the same, except for using 2 μL from the first PCR as the template. The test was considered positive if a 214-bp product was amplified. OCI was defined as the presence of HCV-RNA in PBMCs, with undetectable levels of anti-HCV and HCV-RNA in plasma. The β-actin gene was also amplified as a PCR control for plasma and PMBC samples of each donor, using the same reagents. The primers sequences were 5'-ATGTGGCCGAGGACTTTGATT-3' (forward primer) and 5'-AGTGGGGTGGCTTTTAGGATG-3' (reverse primer). Amplification was performed under the following conditions: initial denaturation at 95 °C for 1 min, followed by 30 cycles of 95 °C for 15 s, 60 °C for 20 s, and 72 °C for 15 s, and a final step of 72 °C for 5 min. A PCR product of 110-bp indicated proper RNA isolation and PCR analysis.

### Serology and laboratory tests

Detection of antibodies against HCV, Hepatitis B Virus (HBV), Human Immunodeficiency Virus (HIV), *Treponema pallidum*, and *Trypanosoma cruzi* was performed by chemiluminescence (CLIA) using the Abbott ARCHITECT Reagent Kit (Ref. 6C37, Abbott, Illinois, USA). The determination of alanine aminotransferase (ALT) was performed on the ARCHITECT c16000 (Abbott).

### Nucleic acid testing (NAT)

The NAT confirmatory test was performed on the Procleix Tigris system (Grifols Diagnostic Solutions Inc. Los Angeles, California, USA) with the Procleix Hrio Assay C.A kit (Gen Probe, San Diego C.A. USA) following the manufacturer’s instructions.

### Statistical analysis

Statistical analyses were performed using Statistical Package for the Social Sciences program, version 21 (SPSS, Chicago, IL). Differences between categorical data were assessed with the Chi-Square test or the Fisher’s exact test. Logistic regression was used to determine the level of association by calculating the Odds Ratio (OR) and 95% confidence interval (95%CI). P-values <0.05 (two-tailed) were considered statistically significant.

## Results

Originally, 1,050 blood donors agreed to participate. Eleven donors were identified to have other comorbidities: 2 were positive for HIV by serology, 3 were positive for anti-HCV but were negative for NAT, 1 was positive for anti-HBV, anti-HIV, and anti-HCV, 2 were positive for *T*. *cruzi*, and 3 donors to *T*. *pallidum*. Thus, these blood samples were removed from the cohort, according to Mexican regulations. Two donors withdrew from the study for unspecified reasons. Of the remaining 1,037 samples, the prevalence of OCI for the study population was 3.4%. The demographic data of the cohort are shown in Table 1. Even though there were almost double the numbers of males (n = 707) than females (n = 330), there was no difference in the distribution of OCI positive cases. When the donors were stratified into age groups, there were no differences in the distribution of OCI positive cases. Interestingly, neither sex nor age was considered factors influencing OCI.

**Table 1:** Characteristics of the study population.

Category	Total	OCI(+)	X^2^/Fisher	Odds Ratio	95% CI	P-value
*Sex*			0.561			
Male	707	24		1.00	Referent	-
Female	330	11		0.98	0.48–2.03	0.954
*Age*			0.655			
18–30	349	14		1.00	Referent	-
31–40	343	13		0.94	0.44–2.04	0.880
41–50	238	6		0.62	0.23–1.63	0.333
51–65	96	2		0.51	0.11–2.28	0.377
*ALT*			0.737			
Normal	953	32		1.00	Referent	-
Elevated	75	3		1.20	0.36–4.03	0.764

Abbreviations: ALT: alanine aminotransferase; OCI: occult Hepatitis C virus infection; and 95% CI: 95% confidence interval. X^2^: Pearson chi-square test. F: Fisher exact test.

* indicates a significant result (p<0.05, two-tailed)

When the donors were separated based on plasma ALT levels, 92.7% had normal ALT levels (<40 IU/L). Of the 75 donors that had elevated levels of ALT, OCI was detected in 3 donors; however, there was no statistical difference in the rate of OCI based on ALT levels (Table 1). Moreover, ALT levels were not a factor influencing OCI, even after adjusting for sex and age (data not shown).

Donors were given a questionnaire to answer to determine the association between lifestyle factors and OCI. However, some categories of the questionnaire were not completed, which is reflected in the smaller sample size. We determined that OCI was significantly associated with homosexual relationships (OR = 5.52, 95%CI: 1.53–19.92, p<0.05) and acupuncture (OR = 3.56, 95%CI: 1.41–8.98, p<0.05, Table 2). Blood donation and transfusion as well as surgeries, piercing, incarceration, colonoscopies, endoscopies, tattoos, and drug use were not associated with OCI. For a complete list, see the S1 Table.

**Table 2:** Lifestyle factors associated with Occult HCV infection.

Risk Factor	Total	OCI(+)	X^2^/Fisher	OR	95% CI	P-value
*Donate Blood*			0.797			
No	753	25		1.00	Referent	-
Yes	274	10		0.90	0.43–1.90	0.786
*Sexual Preference*			0.016*			
Heterosexual	952	31		1.00	Referent	-
Homosexual	20	3		5.52	1.53–19.92	0.009 *
Bisexual	5	0		N/A	N/A	N/A
*Transfusions*			0.378			
No	956	31		1.00	Referent	-
Yes	63	3		1.40	0.42–4.72	0.583
*Surgery*			0.655			
No	641	23		1.00	Referent	-
Yes	391	12		0.85	0.42–1.73	0.654
*Used Drugs*			0.200			
No	901	32		1.00	Referent	-
Yes	86	1		0.32	0.04–2.34	0.259
*Tattoos*			0.237			
No	899	32		1.00	Referent	-
Yes	79	1		0.35	0.05–2.58	0.302
*Piercing*			0.172			
No	220	21		1.00	Referent	-
Yes	230	10		1.56	0.72–3.37	0.255
*Acupuncture*			0.013*			
No	919	27		1.00	Referent	-
Yes	61	6		3.56	1.41–8.98	0.007*
*Incarcerated*			0.559			
No	971	33		1.00	Referent	-
Yes	17	0		N/A	N/A	N/A
*Colonoscopy*			0.460			
No	198	7		1.00	Referent	-
Yes	116	3		0.72	0.18–2.84	0.638
*Endoscopy*			0.554			
No	913	30		1.00	Referent	-
Yes	55	2		1.10	0.26–4.71	0.901

Abbreviations: OCI: occult Hepatitis C virus infection; OR: Odds ratio; and 95% CI: 95% confidence interval. X^2^: Pearson chi-square test. F: Fisher exact test.

* indicates a significant result (p<0.05, two-tailed)

## Discussion

Hepatitis C is a global health problem and transfusion of contaminated blood products is one of the most important mechanisms for its transmission. In Mexico, voluntary donation is rare and more than 90% of the population that goes to a blood bank are family or replacement donors [22]. One of the main objectives of a blood bank is to provide safe, pathogen-free blood. Even though NAT is a highly sensitive molecular technique used to detect plasma HCV-RNA, it is unable to detect the presence of HCV-RNA in PBMCs and consequently OCI. Here, we performed a search for HCV-RNA in plasma and PMBCs using highly sensitive molecular techniques, RT-PCR and nested PCR. With our system, we observed a sensitivity of about 50 genome equivalents (6 IU/mL) in about 70% of patients with OCI [23,24]. The remainder of patients had HCV genome equivalents less than 6 IU/mL, which would present a non-infectious state. Using these techniques, we identified 35 blood donors that were positive for OCI out of 1,037 blood donors that were negative for the detection of serum HCV-RNA or anti-HCV. Therefore, we presumed the OCI prevalence in Mexico City to be 3.4%, which is remarkably higher than the prevalence of HCV-infected persons (1.4%). The Mexican prevalence of OCI is noticeably high, considering that the study population is a random population of individuals, who believe themselves healthy. Our results are similar with reported prevalences in blood donor populations from Egypt (5.7%) [15], China (2.2%) [16] and Spain (2.1%) [17].

It has been reported that only 20 copies of HCV are required for a productive infection [23]. Moreover, OCI has been described as a replicating form of HCV [8]. Normally, blood from a donor is fractionated into 4 blood components by means of separation and leukoreduction techniques; however, the presence of residual leukocytes is inevitable for each unit recovered [24]. Therefore, the non-detection of OCI represents a highly efficient mechanism for the spread of HCV infections. So, we consider it a priority to implement sensitive techniques to identify OCI as part of the screening tests of blood products.

For CHC, elevated ALT is more frequent. In our study, we found that only 8.57% of OCI patients had elevated ALT values. Therefore, we posit that ALT levels are not an important test to indicate a possible OCI [25]. On the other hand, the fact that the majority of OCIs are donors that presented with normal ALT levels, suggests that they may be at an early stage of infection or that the progression to cirrhosis in OCI is lower than in CHC.

A recent report, issued by the World Health Organization, indicates that the number of people infected with the HCV tends to increase with the use of intravenous drugs [2]. At this time, the prevalence of OCI in intravenous drug users is unknown. In contrast to what happens in other countries, in Mexico, the main drug used is marijuana, followed by cocaine [26]. Therefore, it was expected that the use of drugs should not be a risk factor for OCI. Indeed, in our study the use of drugs had no significant association with the presence of OCI.

With HCV infections, homosexual relationships have been described as an important risk factor for transmission; however, for OCI, limited information is available. Here, 2.1% of the study population was homosexual and only 3 donors had OCI. We determined a strong association between homosexual relationships and OCI (OR = 5.52), which suggests that a homosexual relationship is a risk factor. Shazly et al. reported that 4% of healthy spouses of CHC patients have OCI, supporting the notion that sexual transmission of OCI occurs [27]. In the present study, 97.4% were heterosexual and 3.2% of these were positive for OCI, which is in agreement with what was previously reported. Using random sampling, our bisexual group only consisted of 5 individuals, which makes any inference questionable. Here in Mexico, there is no negative stigma regarding the sexual preferences of donors, which suggests that a majority of the participant responded truthfully to the questionnaire.

Acupuncture is a major form of transmission for HCV in countries, such as Korea, where its practice is frequent [28]. However, for OCI, acupuncture has not been shown to be a route for transmission. Here, a significant association was found between the application of acupuncture and OCI (p<0.01). This suggests that acupuncture could be an important route of transmission. In support of this, Lemos et al. demonstrated that the needles used for acupuncture were contaminated with HCV-RNA [29].

In our laboratory, we emphasized the care in collecting a blood sample, its unequivocal identification, and the use of molecular techniques with high sensitivity and specificity in the identification of HCV. Carreño et al. pointed out important factors that have been identified as limiting the detection of OCI [30], which could explain the differences in the prevalence found worldwide. Although the molecular techniques that were used here have a high sensitivity and specificity, they are not implemented by automated equipment that allows to process a large number of samples, which is a requirement in blood banks where there is a large amount to analyze. Up to 1000 samples are processed daily, so faster techniques, such as real-time PCR, must be implemented.

## Conclusion

In Mexico, no previous studies have been conducted to establish the prevalence of OCI in blood donors. Based on our results, we can conclude that the prevalence of OCI in blood donors in Mexico City is high (3.4%) and is not being detected in blood banks by standard procedures. The main risk factors for OCI transmission are homosexual relationships and acupuncture. The detection of OCI in blood banks can significantly reduce the spread of HCV infections, thus significantly reducing medical costs for the care and treatment of patients with CHC. The detection of OCI is important, not only in blood banks but also in molecular biology laboratories, where the presence of HCV is monitored in liver transplant patients, dialysis patients, multi-transfused hemophiliacs, and in patients who, after having reached sustained viral response (SVR), have relapsed [31,32].

## Supporting information

S1 Table Lifestyle factors associated with Occult HCV infection. (DOCX)
