# Supplementary material for: Prevalence and risk factors of Occult Hepatitis C infections in blood donors from Mexico City
Source: PLoS One. 2018 Oct 19;13(10):e0205659. doi: 10.1371/journal.pone.0205659 (PMC6195272; doi:10.1371/journal.pone.0205659)
Supplement: S1 Table — (DOCX) [file pone.0205659.s001.docx]

**S1 Table. Lifestyle factors associated with Occult HCV infection.**

| Risk Factor | Total | OCI(+) | X^2^/Fisher | OR | 95% CI | P-value |
| --- | --- | --- | --- | --- | --- | --- |
| *Occupational Biological Contact* |  |  | 0.109 |  |  |  |
| No | 982 | 31 |  | 1.00 | Referent | - |
| Yes | 55 | 4 |  | 2.43 | 0.83-7.15 | 0.107 |
| *Exposure of Biological Material* |  |  | 0.363 |  |  |  |
| No | 1021 | 34 |  | 1.00 | Referent | - |
| Yes | 13 | 1 |  | 2.61 | 0.33-10.82 | 0.365 |
| *Donate Blood* |  |  | 0.797 |  |  |  |
| No | 753 | 25 |  | 1.00 | Referent | - |
| Yes | 274 | 10 |  | 0.90 | 0.43-1.90 | 0.786 |
| *Number of Blood Donations* |  |  | 0.862 |  |  |  |
| 0 | 262 | 10 |  | 1.00 | Referent | - |
| 1 | 238 | 6 |  | 0.65 | 0.23-1.82 | 0.416 |
| 2 | 177 | 8 |  | 1.19 | 0.46-3.07 | 0.722 |
| 3 | 100 | 3 |  | 0.78 | 0.21-2.88 | 0.703 |
| 4 | 91 | 2 |  | 0.56 | 0.12-2.59 | 0.456 |
| ≥5 | 156 | 6 |  | 0.99 | 0.35-2.79 | 0.988 |
| *Sexual Preference* |  |  | 0.016* |  |  |  |
| Heterosexual | 952 | 31 |  | 1.00 | Referent | - |
| Homosexual | 20 | 3 |  | 5.52 | 1.53-19.92 | 0.009 * |
| Bisexual | 5 | 0 |  | N/A | N/A | N/A |
| *Number of partners* |  |  | 0.087 |  |  |  |
| 1 | 312 | 6 |  | 1.00 | Referent | - |
| 2 | 215 | 5 |  | 1.23 | 0.37-4.08 | 0.738 |
| 3 | 177 | 11 |  | 3.36 | 1.22-9.24 | 0.019 * |
| 4 | 115 | 6 |  | 2.80 | 0.88-8.86 | 0.080 |
| ≥5 | 192 | 7 |  | 1.91 | 0.63-5.75 | 0.253 |
| *Transfusions* |  |  | 0.378 |  |  |  |
| No | 956 | 31 |  | 1.00 | Referent | - |
| Yes | 63 | 3 |  | 1.40 | 0.42-4.72 | 0.583 |
| *Number of transfusion* |  |  | 0.065 |  |  |  |
| 0 | 950 | 31 |  | 1.00 | Referent | - |
| 1 | 41 | 0 |  | N/A | N/A | N/A |
| 2 | 11 | 1 |  | 2.93 | 0.36-23.60 | 0.313 |
| 3 | 5 | 1 |  | 7.32 | 0.80-67.45 | 0.079 |
| 4 | 6 | 1 |  | 5.86 | 0.66-51.65 | 0.111 |
| ≥5 | 6 | 0 |  | N/A | N/A | N/A |
| *Surgery* |  |  | 0.655 |  |  |  |
| No | 641 | 23 |  | 1.00 | Referent | - |
| Yes | 391 | 12 |  | 0.85 | 0.42-1.73 | 0.654 |
| *Number of Surgeries* |  |  | 0.862 |  |  |  |
| 0 | 641 | 23 |  | 1.00 | Referent | - |
| 1 | 221 | 6 |  | 0.75 | 0.30-1.87 | 0.540 |
| 2 | 99 | 3 |  | 0.84 | 0.25-2.85 | 0.778 |
| 3 | 41 | 2 |  | 1.36 | 0.31-5.99 | 0.682 |
| 4 | 12 | 0 |  | N/A | N/A | N/A |
| ≥5 | 14 | 0 |  | N/A | N/A | N/A |
| *Dental Surgery* |  |  | 0.235 |  |  |  |
| No | 648 | 25 |  | 1.00 | Referent | - |
| Yes | 366 | 9 |  | 0.63 | 0.29-1.36 | 0.234 |
| *Number of Dental Surgery* |  |  | 0.247 |  |  |  |
| 0 | 648 | 25 |  | 1.00 | Referent | - |
| 1 | 151 | 2 |  | 0.34 | 0.08-1.43 | 0.139 |
| 2 | 112 | 2 |  | 0.45 | 0.11-1.93 | 0.284 |
| 3 | 45 | 1 |  | 0.56 | 0.07-4.22 | 0.573 |
| 4 | 22 | 2 |  | 2.46 | 0.55-11.11 | 0.242 |
| ≥5 | 30 | 2 |  | 1.76 | 0.40-7.79 | 0.458 |
| *Organ transplant recipient* |  |  | 0.901 |  |  |  |
| No | 1018 | 35 |  | 1.00 | Referent | - |
| Yes | 3 | 0 |  | N/A | N/A | N/A |
| *Family member with HCV* |  |  | 0.901 |  |  |  |
| No | 986 | 35 |  | 1.00 | Referent | - |
| Yes | 47 | 0 |  | N/A | N/A | N/A |
| *Lives with HCV patient* |  |  | 0.901 |  |  |  |
| No | 1005 | 35 |  | 1.00 | Referent | - |
| Yes | 27 | 0 |  | N/A | N/A | N/A |
| *Used Drugs* |  |  | 0.200 |  |  |  |
| No | 901 | 32 |  | 1.00 | Referent | - |
| Yes | 86 | 1 |  | 0.32 | 0.04-2.34 | 0.259 |
| *Type of drug* |  |  | 0.705 |  |  |  |
| None | 903 | 32 |  | 1.00 | Referent | - |
| Marihuana | 63 | 1 |  | 0.43 | 0.06-3.23 | 0.414 |
| Cocaine | 16 | 0 |  | N/A | N/A | N/A |
| Others | 4 | 0 |  | N/A | N/A | N/A |
| *Tattoos* |  |  | 0.237 |  |  |  |
| No | 899 | 32 |  | 1.00 | Referent | - |
| Yes | 79 | 1 |  | 0.35 | 0.05-2.58 | 0.302 |
| *Number of Tattoos* |  |  | 0.843 |  |  |  |
| 0 | 895 | 32 |  | 1.00 | Referent | - |
| 1 | 40 | 0 |  | N/A | N/A | N/A |
| 2 | 26 | 1 |  | 1.11 | 0.15-8.47 | 0.919 |
| 3 | 5 | 0 |  | N/A | N/A | N/A |
| 4 | 3 | 0 |  | N/A | N/A | N/A |
| ≥5 | 7 | 0 |  | N/A | N/A | N/A |
| *Years since first Tattoo* |  |  | 0.843 |  |  |  |
| 0 | 895 | 32 |  | 1.00 | Referent | - |
| 1 | 4 | 0 |  | N/A | N/A | N/A |
| 2 | 7 | 0 |  | N/A | N/A | N/A |
| 3 | 6 | 1 |  | 5.33 | 0.61-46.97 | 0.132 |
| 4 | 3 | 0 |  | N/A | N/A | N/A |
| ≥5 | 63 | 0 |  | N/A | N/A | N/A |
| *Piercing* |  |  | 0.172 |  |  |  |
| No | 220 | 21 |  | 1.00 | Referent | - |
| Yes | 230 | 10 |  | 1.56 | 0.72-3.37 | 0.255 |
| *Number of Piercings* |  |  | 0.787 |  |  |  |
| 0 | 742 | 22 |  | 1.00 | Referent | - |
| 1 | 144 | 6 |  | 1.41 | 0.56-3.53 | 0.469 |
| 2 | 55 | 3 |  | 1.90 | 0.55-6.57 | 0.310 |
| 3 | 13 | 0 |  | N/A | N/A | N/A |
| 4 | 6 | 0 |  | N/A | N/A | N/A |
| ≥5 | 10 | 0 |  | N/A | N/A | N/A |
| *Years since first piercing* |  |  | <0.001* |  |  |  |
| 0 | 743 | 22 |  | 1.00 | Referent | - |
| 1 | 2 | 0 |  | N/A | N/A | N/A |
| 2 | 8 | 0 |  | N/A | N/A | N/A |
| 3 | 5 | 2 |  | 21.58 | 3.43-135.69 | 0.001* |
| 4 | 6 | 0 |  | N/A | N/A | N/A |
| ≥5 | 203 | 7 |  | 1.17 | 0.49-2.77 | 0.725 |
| *Acupuncture* |  |  | 0.013* |  |  |  |
| No | 919 | 27 |  | 1.00 | Referent | - |
| Yes | 61 | 6 |  | 3.56 | 1.41-8.98 | 0.007* |
| *Number of acupuncture sessions* |  |  | 0.009* |  |  |  |
| 0 | 919 | 27 |  | 1.00 | Referent | - |
| 1 | 38 | 4 |  | 3.84 | 1.27-11.59 | 0.017* |
| 2 | 8 | 0 |  | N/A | N/A | N/A |
| 3 | 3 | 0 |  | N/A | N/A | N/A |
| 4 | 2 | 0 |  | N/A | N/A | N/A |
| ≥5 | 10 | 2 |  | 8.16 | 1.65-40.25 | 0.010* |
| *Years of acupuncture* |  |  | <0.001* |  |  |  |
| 0 | 920 | 27 |  | 1.00 | Referent | - |
| 1 | 11 | 3 |  | 12.25 | 3.08-48.74 | <0.001* |
| 2 | 4 | 1 |  | 10.89 | 1.10-108.10 | 0.041* |
| 3 | 5 | 0 |  | N/A | N/A | N/A |
| 4 | 2 | 0 |  | N/A | N/A | N/A |
| ≥5 | 38 | 2 |  | 1.82 | 0.42-7.93 | 0.428 |
| *Incarcerated* |  |  | 0.559 |  |  |  |
| No | 971 | 33 |  | 1.00 | Referent | - |
| Yes | 17 | 0 |  | N/A | N/A | N/A |
| *Psychiatric Hospital* |  |  | 0.843 |  |  |  |
| No | 978 | 33 |  | 1.00 | Referent | - |
| Yes | 5 | 0 |  | N/A | N/A | N/A |
| *Ingrown Nails* |  |  | 0.213 |  |  |  |
| No | 861 | 31 |  | 1.00 | Referent | - |
| Yes | 119 | 2 |  | 0.46 | 0.11-1.93 | 0.286 |
| *Sharing razors* |  |  | 0.216 |  |  |  |
| No | 908 | 28 |  | 1.00 | Referent | - |
| Yes | 74 | 4 |  | 1.77 | 0.61-5.20 | 0.296 |
| *Colonoscopy* |  |  | 0.460 |  |  |  |
| No | 198 | 7 |  | 1.00 | Referent | - |
| Yes | 116 | 3 |  | 0.72 | 0.18-2.84 | 0.638 |
| *Number of Colonoscopy* |  |  | 0.653 |  |  |  |
| 0 | 901 | 27 |  | 1.00 | Referent | - |
| 1 | 40 | 0 |  | N/A | N/A | N/A |
| 2 | 24 | 1 |  | 1.26 | 0.17-9.66 | 0.823 |
| 3 | 16 | 1 |  | 1.85 | 0.24-14.45 | 0.558 |
| 4 | 14 | 1 |  | 2.13 | 0.27-16.83 | 0.472 |
| ≥5 | 23 | 0 |  | N/A | N/A | N/A |
| *Endoscopy* |  |  | 0.554 |  |  |  |
| No | 913 | 30 |  | 1.00 | Referent | - |
| Yes | 55 | 2 |  | 1.10 | 0.26-4.71 | 0.901 |
| *Number of Endoscopy* |  |  | 0.007* |  |  |  |
| 0 | 910 | 30 |  | 1.00 | Referent | - |
| 1 | 35 | 1 |  | 0.88 | 0.12-6.64 | 0.900 |
| 2 | 10 | 0 |  | N/A | N/A | N/A |
| 3 | 5 | 0 |  | N/A | N/A | N/A |
| 4 | 2 | 1 |  | 29.00 | 1.77-474.80 | 0.018* |
| *Vaginal exams with disposable equipment* |  |  | 0.261 |  |  |  |
| No | 107 | 2 |  | 1.00 | Referent | - |
| Yes | 200 | 8 |  | 2.17 | 0.45-10.40 | 0.333 |

X^2^: Pearson chi-square test

F: Fisher exact test

* indicates a significant result (p<0.05, two-tailed)
